# Quenching corrinoid-based interactions in a model bacterial coculture

**DOI:** 10.1093/ismeco/ycag160

**Published:** 2026-06-09

**Authors:** Zachary F Hallberg, Zoila I Alvarez-Aponte, Allison Gaudinier, Michiko E Taga

**Affiliations:** Department of Plant & Microbial Biology, University of California, Berkeley, Berkeley, CA 94720, United States; Department of Plant & Microbial Biology, University of California, Berkeley, Berkeley, CA 94720, United States; Department of Plant & Microbial Biology, University of California, Berkeley, Berkeley, CA 94720, United States; Department of Plant & Microbial Biology, University of California, Berkeley, Berkeley, CA 94720, United States

**Keywords:** corrinoids, vitamin B_12_, microbial ecology, cross-feeding, nutritional immunity

## Abstract

Microbial community structure is driven, in part, by the metabolic interdependencies of resident microbes. Thus, manipulating specific metabolic interactions represents an attractive way to both understand how microbial communities perform complex functions and alter them for therapeutic or environmental effects. However, it is not yet possible to control the availability of those metabolites produced by some members of the community that are required by others. Here, we report the development of a metabolite “quenching” strategy that disrupts a specific metabolic interaction involving corrinoids, the vitamin B_12_ family of cofactors, by applying a high-affinity corrinoid-binding protein, BtuG, to bacteria engaged in corrinoid cross-feeding. Using a model coculture composed of *Sinorhizobium meliloti*, a bacterium that produces a corrinoid (cobalamin), and an *Escherichia coli* strain engineered to be corrinoid-dependent, we demonstrate corrinoid quenching by sequestration of extracellular corrinoid, leading to inhibition of corrinoid-dependent growth. This work establishes a strategy to selectively block microbial interactions that may be more broadly applied to dissecting community structure and function. We expect that applying high-affinity “molecular sponges” to quench nutrient sharing will allow for the identification of key nutrients that structure microbial communities and potentiate precision microbiome manipulation strategies.

Nutrient competition and cross-feeding shape the function and composition of microbial communities [1, 2]. Identifying nutrients that are key to structuring a given microbial community is critical to understanding how these ecological processes contribute to community composition. However, while altering the availability of an externally supplied nutrient such as a carbon source is straightforward, it remains difficult to control the availability of nutrients produced by and shared within a community. To address this limitation, we took inspiration from natural examples where metabolite availability is disrupted, or “quenched.” For example, in nutritional immunity, a host uses a high-affinity binding protein to sequester iron to limit pathogen growth [3, 4]. Furthermore, some bacteria degrade quorum-sensing autoinducer signals to inhibit group behavior in competing microbes, a process termed quorum quenching [5]. Developing methods to quench the availability of other metabolites, such as biosynthesized cofactors, would provide a powerful strategy to dissect nutrient interdependence and selectively control the growth of beneficial or pathogenic microbes.

Here, we describe a technique to quench interactions involving a family of biosynthesized micronutrients, corrinoids—the vitamin B_12_ (cobalamin) family of enzyme cofactors. Genomic data predict that corrinoids are synthesized by only 36% of bacterial species, but used by over 80% [6–9], suggesting that they are important shared metabolites in microbial communities. These predicted corrinoid-mediated interactions are prevalent across microbial ecosystems of import, ranging from the production of health-promoting metabolites in the human gut to the growth of marine phytoplankton [1, 10]. We and others have shown that corrinoid addition to microbial communities influences community structure [11–14], demonstrating that corrinoids have the ability to alter the composition of microbial communities. Nevertheless, direct demonstration of their importance as naturally shared metabolites in a community context remains lacking, and could be performed with a suitable corrinoid quenching method. To demonstrate that corrinoid interactions can be quenched, we used BtuG, a femtomolar-affinity protein that binds corrinoids with a 1:1 stoichiometry and is required for corrinoid uptake in *Bacteroides thetaiotaomicron* [15, 16]. This quenching method offers a proof-of-concept that can be applied to manipulate interactions involving other shared nutrients.

We first established a model coculture system consisting of corrinoid-producing and -dependent bacteria. As the producer, we selected *Sinorhizobium meliloti* (*Sm*) because unlike other bacteria such as *Pseudomonas denitrificans*, it synthesizes and releases corrinoids (specifically cobalamin) into the extracellular environment (Fig. 1a) for potential use by corrinoid-dependent microbes in coculture (Fig. 1b). We use “release” to describe any potential mechanism by which a corrinoid becomes extracellular, whether through active secretion, passive diffusion, or cell death. For the corrinoid-dependent strain, we used an *Escherichia coli ΔmetE* mutant (abbreviated as *Ec*), which relies on a corrinoid for growth when no methionine is supplied [17, 18]. To enable specific detection of *Ec* in mixed cocultures, this strain was engineered to constitutively express green fluorescent protein. To establish the growth response of the two species in mono- and coculture, we measured O.D._600_ and fluorescence. We screened multiple carbon sources for the ability to support growth of the two species (Fig. 1c and d). While many carbon sources supported the growth of both species in monoculture (Groups 1, 2), others supported growth of only one species in monoculture (Groups 3, 4). Coculture growth was only observed in a subset of the carbon sources that support monoculture growth of both species (Groups 1, 2). We selected glycerol as the carbon source for subsequent experiments, as neither species exhibits a distinct growth advantage in this medium, and *E. coli* does not release acetate when grown in this carbon source, which could act as an additional cross-fed nutrient [19].

**Figure 1:**
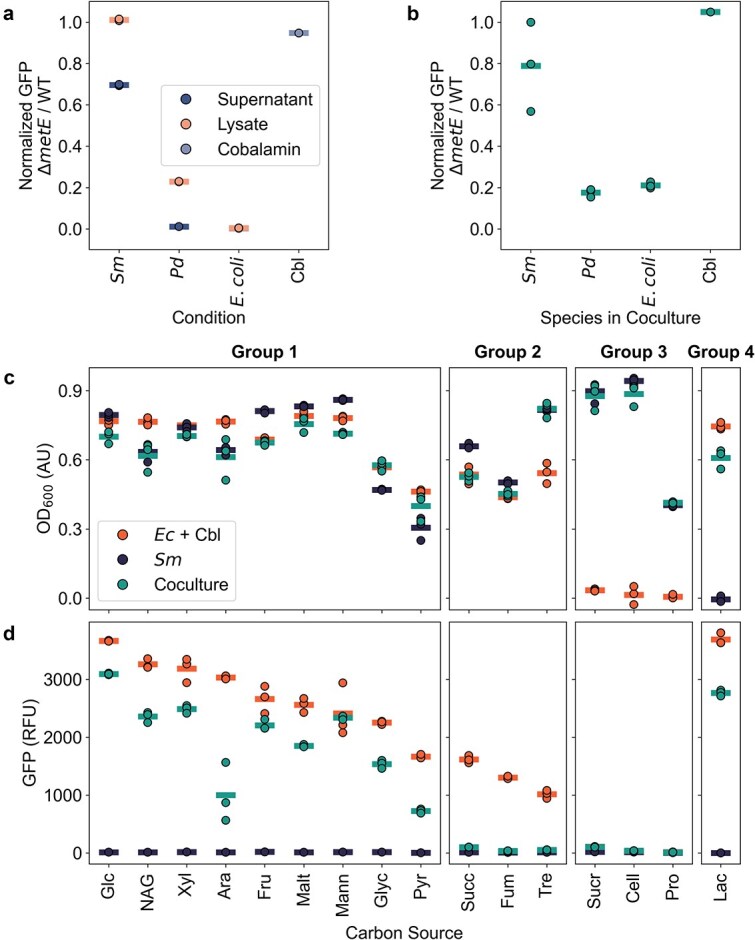
Growth of *Ec* in monoculture and in coculture with *Sm*. (a) GFP fluorescence (in relative fluorescence units) of *Ec gfp^+^* monocultures in the presence of supernatant or cell lysate of wild type corrinoid producers *Sm* and Pd, and of non-producer *E. coli. Pd* is included as a control corrinoid-producing species that does not release corrinoid. (b) GFP fluorescence of cocultures containing *Ec* and *Sm*. Values on the y-axis in panels a and b represent the ratio of the GFP signal produced by *Ec* to the GFP signal of the parental *gfp^+^ E. coli* strain grown under the same conditions. (c) O.D._600_ (absorbance units) and (d) GFP fluorescence at 48 h are shown for *Sm* in monoculture, *Ec* supplemented with cyanocobalamin (vitamin B_12_) in monoculture, and cocultures of both grown in M9 medium with the indicated carbon source. The data are grouped by phenotype: Group 1—carbon sources in which both species grow in monoculture and *Ec* grows in coculture, Group 2—carbon sources in which both species grow in monoculture but *Ec* does not grow in coculture. Groups 3 and 4 represent carbon sources in which only *Sm* (Group 3) or *Ec* (Group 4) can grow in monoculture. Values for uninoculated controls were subtracted from raw O.D._600_ and fluorescence measurements. Average values for triplicate biological samples are shown as horizontal lines. Carbon sources are abbreviated as: Ara, arabinose; AU, absorbance units; Cell, cellobiose; Fru, fructose; Fum, fumarate; GFP; green fluorescent protein; Glc, glucose; Glyc, glycerol; Lac, lactose; Malt, maltose; Mann, mannitol; NAG, N-acetylglucosamine; Pd, *P. denitrificans*; Pro, proline; Pyr, pyruvate; RFU, relative fluorescence units; Succ, succinate; Sucr, sucrose; Tre, trehalose; Xyl, xylose.

To test specificity of BtuG as a corrinoid quencher, we first determined whether the addition of purified BtuG protein to *Ec* grown in monoculture could specifically inhibit corrinoid-dependent growth. When cultured in 1 nM cobalamin with increasing concentrations of purified BtuG, we found that growth was inhibited at concentrations higher than 1.23 nM (Fig. 2a). This BtuG-dependent growth suppression is specific to cobalamin metabolism, because when methionine is supplied instead of cobalamin, growth remains robust at high BtuG concentrations (Fig. 2a).

**Figure 2:**
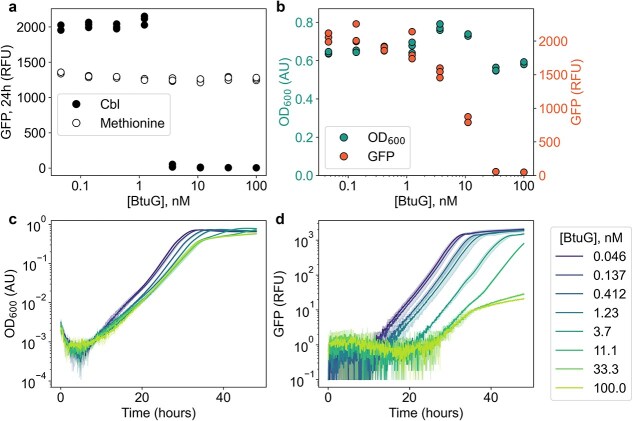
Mono- and coculture response to corrinoid quenching. (a) GFP signal (RFU) of *Ec* monocultures supplemented with methionine (1 mg/ml) or cobalamin (1 nM) and increasing concentrations of purified BtuG. The lower GFP fluorescence observed with methionine supplementation may be a result of changes in queuosine biosynthesis, a known corrinoid-dependent pathway in *E. coli* [6, 28]. (b) O.D._600_ (AU) and GFP signal after 48 h from cocultures of *Sm* and *Ec* with increasing concentrations of BtuG. *Ec* is in the only strain expressing GFP. (C and D) Growth curves with O.D._600_ (c) and GFP fluorescence (d) of cocultures of *Sm* and *Ec* supplemented with a range of concentrations of BtuG. Values for uninoculated controls were subtracted from raw O.D._600_ and fluorescence measurements and normalized to an initial OD_600_ of 0.001 (c) and an initial GFP signal of 1 (D). AU, absorbance units; GFP, green fluorescent protein; RFU, relative fluorescence units.

We next tested whether BtuG could affect *Ec* growth when cobalamin is provided by *Sm* in coculture. Indeed, we observed that, when cocultured with *Sm* in media without added cobalamin, *Ec* growth was suppressed by addition of BtuG, with higher concentrations completely inhibiting the growth of *Ec* (Fig. 2b–d). Further, increasing the BtuG concentration above that required to inhibit *Ec* growth does not affect *Sm* growth, confirming that the BtuG treatment specifically targets corrinoid availability. This result provides a proof-of-concept that BtuG can be used as a tool to block corrinoid sharing interactions.

While the use of BtuG permits quenching of corrinoid interactions in our model coculture, potential limitations must be considered for broader microbiome applications. First, the effectiveness of corrinoid quenching depends on the stability of the quenching agent; secreted microbial proteases in multispecies communities could act to degrade BtuG. For example, applications in gut microbial communities could be impacted by BtuG instability, as both the host and microbiome secrete high levels of nonselective proteases [20, 21]. Second, we expect that quenching strategies analogous to our BtuG sequestration method become inefficient with micronutrients that are found at micromolar levels and higher, because stoichiometric equivalents of quencher are required for this strategy. Third, because multiple corrinoid structures exist in most microbial communities [22, 23], and BtuG binds a wide range of them, we expect that BtuG treatment would broadly disrupt corrinoid-based interactions rather than selectively targeting interactions involving a specific corrinoid. Nonetheless, titration of BtuG concentrations could be used to distinguish between dependent organisms with different transporter affinities [16, 24].

Here, we have developed a strategy to sequester corrinoids using a high-affinity corrinoid-binding protein, demonstrating an extracellular corrinoid “sharing” interaction between *Sm* and *E*c in coculture. Our finding that *B. thetaiotaomicron* BtuG can quench a corrinoid-sharing interaction suggests that *Bacteroides* may use BtuG both for corrinoid uptake and potentially to engage in corrinoid quenching as a mechanism to compete with other microbes in the gut [16, 25]. The nutrient quenching technique developed here offers a strategy to investigate metabolite cross-feeding interactions more broadly. Other high-affinity metabolite-binding proteins could be developed as a general tool to investigate the roles of diverse nutrients in shaping microbial community structure, further enabling precision modulation of microbial communities [26, 27].

## Supplementary Material

Hallberg_etal_2025_BtuG_FigureData_Code_ycag160

## Data Availability

All raw data and associated code are available in the supplementary files.
