# Supplementary material for: Quenching corrinoid-based interactions in a model bacterial coculture
Source: ISME Commun. 2026 Jun 9;6(1):ycag160. doi: 10.1093/ismeco/ycag160 (PMC13374852; doi:10.1093/ismeco/ycag160)
Supplement: Hallberg_etal_2025_BtuG_FigureData_Code_ycag160 [file hallberg_etal_2025_btug_figuredata_code_ycag160.zip › Supplemental_BtuG_ycag160.pdf]

Materials and Methods

Strain construction

E. coli strain MG1655 *ΔmetE ΔnfsA::RFP-GFP-kan*^{R} was constructed by P1 transduction of the *ΔnfsA::RFP-GFP-kan*^{R} from MG1655 *ΔnfsA::RFP-GFP-kan*^{R} [1] into *E. coli* strain MG1655 *ΔmetE* [2].

BtuG expression and purification

A signal sequence truncation mutant of BtuG2 from *Bacteroides thetaiotaomicron* VPI-5482 with a C-terminal His_{10} tag in a pET21-derived plasmid was expressed and purified as previously reported [3, 4]. Protein aliquots were diluted in storage buffer (1×PBS, pH 8.0, composed of 10.1 mM Na_2HPO_4, 1.8 mM KH_2PO_4, 137 mM NaCl, 2.7 mM KCl) to a 10x working concentration for experiments.

Strain preparation and preculturing

For preparation of cells for all experiments, strains were streaked from frozen (-80°C) 25% glycerol stocks (*E. coli*, *P. denitrificans* [5]) or 10% DMSO stocks (*S. meliloti* [6]) onto LB-agar (1.5%) plates to obtain single colonies. Individual colonies were inoculated into M9-glucose (0.2%) medium (supplemented with 1× SL10 minerals and 1× Wolin's vitamins with B_{12} omitted). For *E. coli ΔmetE* strains, this modified M9 medium was further supplemented with methionine (1 mg/mL) [7-10]. Cultures were incubated at 30°C for 48 h with shaking at 200 revolutions per minute (rpm). Cells were pelleted by centrifugation (5000 × rcf, 4 min) of 1 mL of saturated culture, the supernatant aspirated, and resuspended in 1× PBS (1 mL, pH 7.4). Cells were washed a total of three times in this way.

Microbial Growth Assays

All growth assays were conducted in 96-well microtiter plates (Corning Costar Assay Plate 3904) and sealed with air-permeable membranes (Breathe-Easy, Diversified Biotech). Cultures were incubated at 30°C for 24-48 h, either in a bench top heated plate shaker (Southwest Science) at 1,200 rpm, or a Biotek Synergy 2 plate reader with linear shaking at 1,140 cycles per minute. Optical density at 600 nm ($O.D._{600}$) and GFP fluorescence (excitation: 485/20, emission: 528/20) were measured in the Biotek Synergy 2 plate reader. Data were plotted and analyzed in Python.

For initial coculture screens, 2 μL of *Sm* culture and 2 μL of *Ec* (if applicable) were added to 200 μL of modified M9 medium (containing SL10 minerals and Wolin's vitamins as above) or supplemented with 1× of the 19 amino acids used for EZ-rich medium [11]. For coculture experiments screening carbon sources, cells were resuspended and combined in M9 medium lacking a carbon source so that both strains were at a starting $O.D._{600}$ of 0.001. 180 μL of this cell mixture was added to 20 μL of carbon source (2% w/v, for a 0.2% final concentration). For controls, 20 μL of 1× PBS was used as a negative control, and 20 μL of 10 nM cyanocobalamin was added for the B_{12} control (final concentration 1 nM). To determine BtuG quenching efficacy, 2 μL of washed *E. coli* culture (diluted to an $O.D._{600}$ of 0.1 in 1× PBS) were added to 180 μL of modified M9-glycerol (0.4%) medium (supplemented with either methionine (1 mg/mL) or vitamin B_{12} (1 nM)) and 20 μL of BtuG at a 10× concentration. For BtuG coculture experiments, 2 μL of prepared *S. meliloti* cells and 2 μL of prepared *E. coli* auxotroph cells (each diluted to an $O.D._{600}$ of 0.1 in 1× PBS) were added to 180 μL of modified M9-glycerol (0.4%) and 20 μL of BtuG at a 10× stock concentration.

Supplemental Table 1. List of bacterial strains used in this study.

Species	Strain	Genotype	Reference
E. coli	MG1655 *ΔmetE*	*ΔmetE::FRT*	[2]
E. coli	MG1655 *ΔmetE nfsA::RFP-GFP-kan*	*ΔmetE::FRT nfsA::P_{J23119}-RFP-P_{J23119}-GFP-kan*	This work
E. coli	MG1655 *nfsA::RFP-GFP-kan*	*nfsA::P_{J23119}-RFP-P_{J23119}-GFP-kan*	[1]
E. coli	BL21(DE3) pET21-BtuG		[3]
S. meliloti	Rm1021		[6]
P. denitrificans	ATCC 13867		[5]

Bibliography

1. Qi LS, Larson MH, Gilbert LA, Doudna JA, Weissman JS, Arkin AP, Lim WA. Repurposing CRISPR as an RNA-guided platform for sequence-specific control of gene expression. Cell. 2013;152(5):1173-83.
2. Mok KC, Hallberg ZF, Taga ME. Purification and detection of vitamin B_{12} analogs. Methods Enzymol. 2022;668:61-85.
3. Wexler AG, Schofield WB, Degnan PH, Folta-Stogniew E, Barry NA, Goodman AL. Human gut Bacteroides capture vitamin B(12) via cell surface-exposed lipoproteins. Elife. 2018;7:e37138.
4. Hallberg ZF, Nicolas AM, Alvarez-Aponte ZI, Mok KC, Sieradzki ET, Pett-Ridge J, et al. Soil microbial community response to corrinoids is shaped by a natural reservoir of vitamin B12. ISME J. 2024;18(1):wrae094.
5. Ainala SK, Somasundar A, Park S. Complete Genome Sequence of Pseudomonas denitrificans ATCC 13867. Genome Announc. 2013;1(3):e00257-13.
6. Meade HM, Long SR, Ruvkun GB, Brown SE, Ausubel FM. Physical and genetic characterization of symbiotic and auxotrophic mutants of *Rhizobium meliloti* induced by transposon Tn5 mutagenesis. J Bacteriol. 1982;149(1):114-22.
7. Wolin EA, Wolin MJ, Wolfe RS. Formation of Methane by Bacterial Extracts. J Biol Chem. 1963;238:2882-6.
8. Widdel F, Pfennig N. Studies on dissimilatory sulfate-reducing bacteria that decompose fatty acids. I. Isolation of new sulfate-reducing bacteria enriched with acetate from saline environments. Description of *Desulfobacter postgatei* gen. nov., sp. nov. Arch Microbiol. 1981;129(5):395-400.
9. Sambrook J, Russell DW. Molecular cloning : a laboratory manual. 3rd ed. Cold Spring Harbor, N.Y.: Cold Spring Harbor Laboratory Press; 2001.
10. Miller JH. Experiments in molecular genetics. Cold Spring Harbor, N.Y.: Cold Spring Harbor Laboratory; 1972.
11. Neidhardt FC, Bloch PL, Smith DF. Culture medium for enterobacteria. J Bacteriol. 1974;119(3):736-47.
